# Improvement of eukaryotic protein predictions from soil metagenomes

**DOI:** 10.1038/s41597-022-01420-4

**Published:** 2022-06-16

**Authors:** Carole Belliardo, Georgios D. Koutsovoulos, Corinne Rancurel, Mathilde Clément, Justine Lipuma, Marc Bailly-Bechet, Etienne G. J. Danchin

**Affiliations:** 1 Institut Sophia Agrobiotech, Université Côte d’Azur, INRAE, CNRS, Sophia Antipolis, France; 2 MYCOPHYTO, 540 Avenue de la Plaine, 06250 Mougins, France

**Keywords:** Soil microbiology, Data processing, Sequence annotation

## Abstract

During the last decades, metagenomics has highlighted the diversity of microorganisms from environmental or host-associated samples. Most metagenomics public repositories use annotation pipelines tailored for prokaryotes regardless of the taxonomic origin of contigs. Consequently, eukaryotic contigs with intrinsically different gene features, are not optimally annotated. Using a bioinformatics pipeline, we have filtered 7.9 billion contigs from 6,872 soil metagenomes in the JGI’s IMG/M database to identify eukaryotic contigs. We have re-annotated genes using eukaryote-tailored methods, yielding 8 million eukaryotic proteins and over 300,000 orphan proteins lacking homology in public databases. Comparing the gene predictions we made with initial JGI ones on the same contigs, we confirmed our pipeline improves eukaryotic proteins completeness and contiguity in soil metagenomes. The improved quality of eukaryotic proteins combined with a more comprehensive assignment method yielded more reliable taxonomic annotation. This dataset of eukaryotic soil proteins with improved completeness, quality and taxonomic annotation reliability is of interest for any scientist aiming at studying the composition, biological functions and gene flux in soil communities involving eukaryotes.

## Abstract (summary)

**Table:** 

Measurement(s)	gene prediction objective
Technology Type(s)	Bioinformatics
Sample Characteristic - Organism	Eukaryota
Sample Characteristic - Environment	bulk soil • rhizosphere • rhizosphere environment
Sample Characteristic - Location	world

## Background & Summary

Soil-dwelling microorganisms play essential biological functions related to human and Earth health in both managed and natural ecosystems^1^. In recent years, the rise of metagenomics has expanded our understanding of the genetic diversity of microorganisms in many different complex environments, including soil and plant-associated microbiomes^2^. Metabarcoding and shotgun metagenomic sequencing have highlighted the high diversity of microbial communities and allowed the discovery of previously unknown microorganisms^3,4^. Recent efforts have focused on the *de novo* assembly of bulk metagenomic sequencing reads into metagenome-assembled genomes (MAGs) or contigs, uncovering the genetic content and informing on the molecular functions of these microorganisms^5–7^.

The soil is arguably one of the most complex microbiome due to the extremely high diversity of organisms, their complex inter-kingdom interactions and the wide spectrum of environmental conditions observed between samples. In comparison, the human gut microbiome is more homogeneous among individuals due to more stable physiological conditions. Therefore, the soil contains many microbial guilds which cover all different superkingdoms of life with disparate metabolic abilities^8^. Most metagenomic studies are focused on bacteria, which dominate microbiome in number of individuals, although eukaryotes often account for a comparable biomass in soils^2^. The composition and diversity of eukaryotic microorganisms in soils are expected to be higher than, and different, from other ecosystems but are still mostly unknown^9–11^. Moreover, eukaryotic soil microorganisms fulfill essential functions in ecosystems, mainly by participating in the biochemical balance^12^ and nutrient cycling^13^. They also affect the biodiversity and health of macro-organisms constituting fauna and the flora. Some eukaryotes are pathogens of plants or animals, and can cause tremendous health or economic damages^14^. In contrast, some others are beneficial such as mycorrhizal fungi which live symbiotically with 90% of the vascular plants on Earth^15^. The mutualistic interactions of plants with eukaryotic microorganisms from the rhizosphere provide them nutritive and protective benefits, giving those fungi a strong agronomic and environmental interest^16–19^.

Despite their prime importance in diverse processes, soil eukaryotes are neglected and not well represented in public metagenomic data. Previous studies have highlighted the poor representation of eukaryotes in standard metagenomics analyses in different environmental samples and proposed strategies to mitigate this under-representation^20,21^. The largest publicly available resource for soil metagenomes is the Integrated Microbial Genomes & Microbes (IMG/M) database of the Joint Genome Institute (JGI)^22^. In this resource, standard pipelines are used to assemble and annotate contigs and genomes from environmental metagenomic shotgun reads. One major limitation concerns the eukaryotic component of these soil metagenomes. Indeed, the gene prediction tool used by default for all contigs assembled from metagenomes is Prodigal^23^, a software tailored for prokaryotes. However, gene structures and features are different in eukaryotes, and using prokaryotic tools to predict eukaryotic genes can lead to incomplete, erroneous and discontinuous gene sequences, and hence proteins: a trivial example is that no intron can be predicted by Prodigal. These procedures make sense given the volume of metagenomic data processed by IMG/M, but, as a consequence, eukaryotic proteins are neglected in these soil microbiome data, with a risk of being truncated and assigned an unreliable taxonomic annotation. These suboptimal sequences and taxonomic annotations then negatively impact any research on the eukaryotic component of the soil.

To circumvent this problem, we have constituted a dataset of 6,872 soil microbiomes comprising 7.9 billion contigs and identified eukaryotic contigs using a k-mer based approach. On the identified eukaryotic contigs, we re-predicted ca. 93 million genes and proteins using annotation methods tailored for eukaryotes. We re-assigned taxonomic information to these proteins based on a last common ancestor (LCA) approach from homology search against the NCBI’s nr library. This allowed identifying 8 million eukaryotic proteins and more than 300,000 orphan proteins located on eukaryotic contigs and lacking homology in public protein libraries, representing a potential for new discoveries. We show that the newly predicted proteins are longer and constitute a more comprehensive representation of the pool of eukaryotic proteins in the soil.

This new dataset improves eukaryotic protein sequence quality and completeness, as well as the reliability of the taxonomic information, and represents a unique resource to decipher and study the pool of eukaryotic proteins present in the soil.

## Methods

### Data collection

**Fig. 1:**
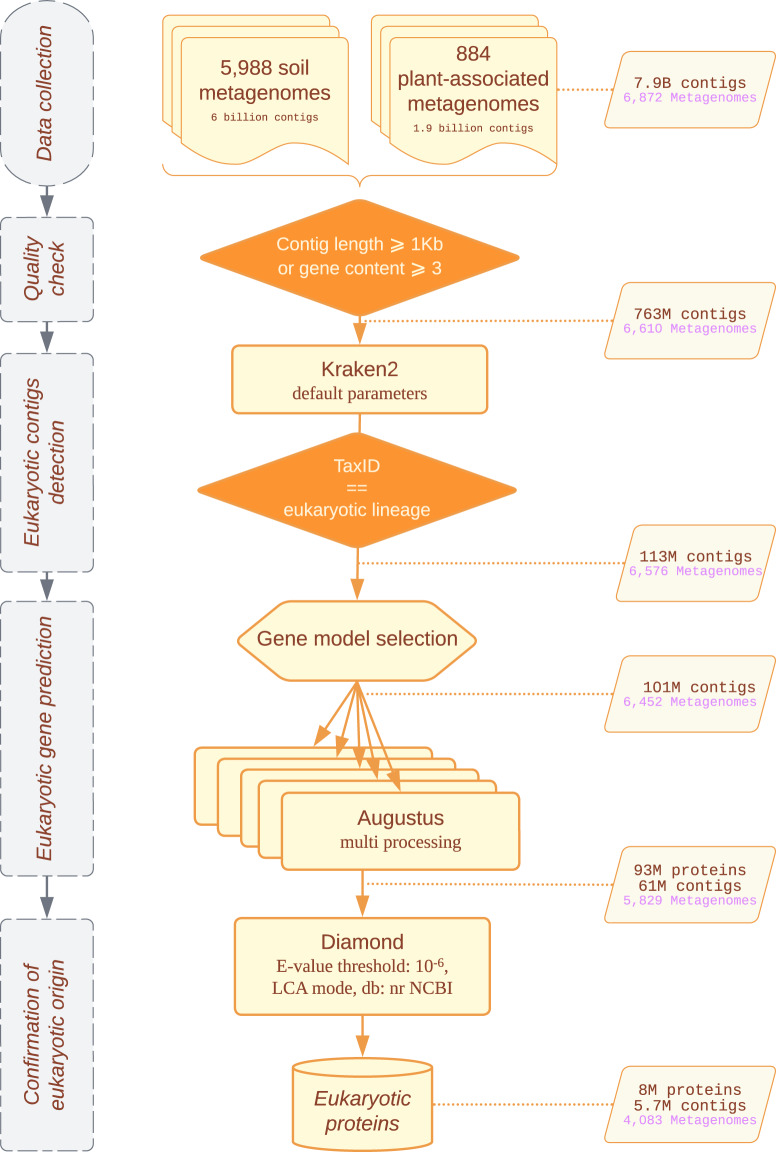
Our eukaryotic protein prediction pipeline from soil metagenomic contigs to a final dataset of taxonomically annotated proteins with contigs, proteins and metagenomes number at each step.

We used publicly available assembled metagenomic data from shotgun sequencing reads of the IMG/M database of the JGI^22^. We collected metagenomes of 5,988 ‘Terrestrial’ samples in the environmental metagenomes category and 884 plant-associated metagenomes in the host-associated category, Fig. 1 (available data 2020, October; Supplementary Data^24^). Most of the datasets were unrestricted from use, according to the JGI policy; the authors of a few datasets (see Acknowledgements) that were still under use-restriction kindly authorized us to re-use their data, including two published in the literature^25,26^. The data acquisition was performed via the IMG/MER Cart genome portal. For each metagenome, the JGI provides a set of files from pre-computed analyses that are useful to sort, filter and describe data. Because we anticipated substantial differences in the relative proportions of eukaryotic species present in the terrestrial and the host-associated categories, these two datasets were processed separately to minimize potential biases. For a more convenient processing of the massive amount of data, the metagenomes from terrestrial samples were splitted in two batches; ‘Terrestrial 1’ contained 3,601 environmental metagenomes added between December 2009 and January 2019 and ‘Terrestrial 2’ contained 2,387 metagenomes added between February 2019 and August 2020.

### Data curation and quality control

Starting from assembled contigs, we combined all genomic fasta files by datasets and obtained 6 and 1.9 billion contigs from terrestrial and plant-associated categories, respectively. The length distribution of assembled contigs is highly heterogeneous between metagenomes due to variation in sequencing technologies, experimental protocols, pipeline version used and biological features. Probably because most data initially consisted of short sequencing reads, half of the contigs were shorter than 296 bp (Table 1). These short contigs increase the volume to be processed and are unlikely to contain complete genes, hence providing no more information on gene diversity^27^. Thus, we filtered data on assembly length, and kept contigs at least 1 kb long or containing at least three genes predicted by Prodigal in JGI files. Only 763 million contigs (10%) passed this filter and were retained for further analysis (Fig. 1). These remaining contigs were distributed in 6,610 metagenomes (Fig. 1): hence data from 262 starting metagenomes were entirely removed due to a too high level of fragmentation. This quality filtering drastically reduced the dataset volume and ensured we only worked with contigs on which complete eukaryotic genes have a chance to be predicted.

**Table 1:** Metrics to assess the contiguity of the 6,872 ‘Terrestrial’ and ‘Plant-associated’ metagenome-assembled genomes datasets from the IMG/M server of the JGI including the number of proteins predicted by Prodigal from IMG/M.

Data	Metric	Min	Mean	Median	Max
Raw	Number of contigs per metagenomes	1	1,160,141	294,105	39,582,895
	Contig length (pb)	3	497	296	5,373,015
	Number of genes per contig	0	1	1	5,459
Filtered	Number of contigs per metagenomes	1	115,615	22,307	3,625,639
	Contig length (pb)	1,000	1,985	1,350	5,373,015
	Number of genes per contig	1	3	2	5,459

### Detection of contigs from eukaryotic organisms

The JGI provides taxonomic information for genes predicted using Prodigal, which is not suitable for eukaryotic genes. Moreover this taxonomic information has been transfered solely from that of the best BLAST hit, which can be misleading. Thus, this information cannot be used to identify potential eukaryotic contigs and no further taxonomic information is provided for contigs. Therefore, we scanned all the contigs and identified those from eukaryotic origins using Kraken2^28^, a taxonomic classification tool based on exact kmer matches, designed to process in a fast and sensitive way large data sets such as those from metagenomics analyses. Among the taxonomic classifiers dedicated to metagenomic data, we selected Kraken2 because it provides taxonomically labeled contigs and it is designed to work on reads but can also process contigs. As a consequence, this software maintains a good sensitivity on short sequences, representing an ideal choice in our case. Indeed, as indicated in Table 1, our data mainly contains short contigs with an average size of 1.9Kb, which would be sub-optimal for usage with a contig-centered software such as Eukrep^20^,that performs better on contigs at least 3Kb long, as mentioned by the authors. As a reference database, we combined all RefSeq libraries of complete genomes [Archaea, Bacteria, Plasmid, Viral, Human, Fungi, Plant, Protozoa]^29^, complemented by the NCBI’s nt library and ran Kraken2 with default parameters. This allowed assigning taxonomic information to 82% of contigs, among which 113 million were classified with a eukaryotic taxonomic identifier ‘TaxID’ (Fig. 1).

### Eukaryotic gene prediction

**Fig. 2:**
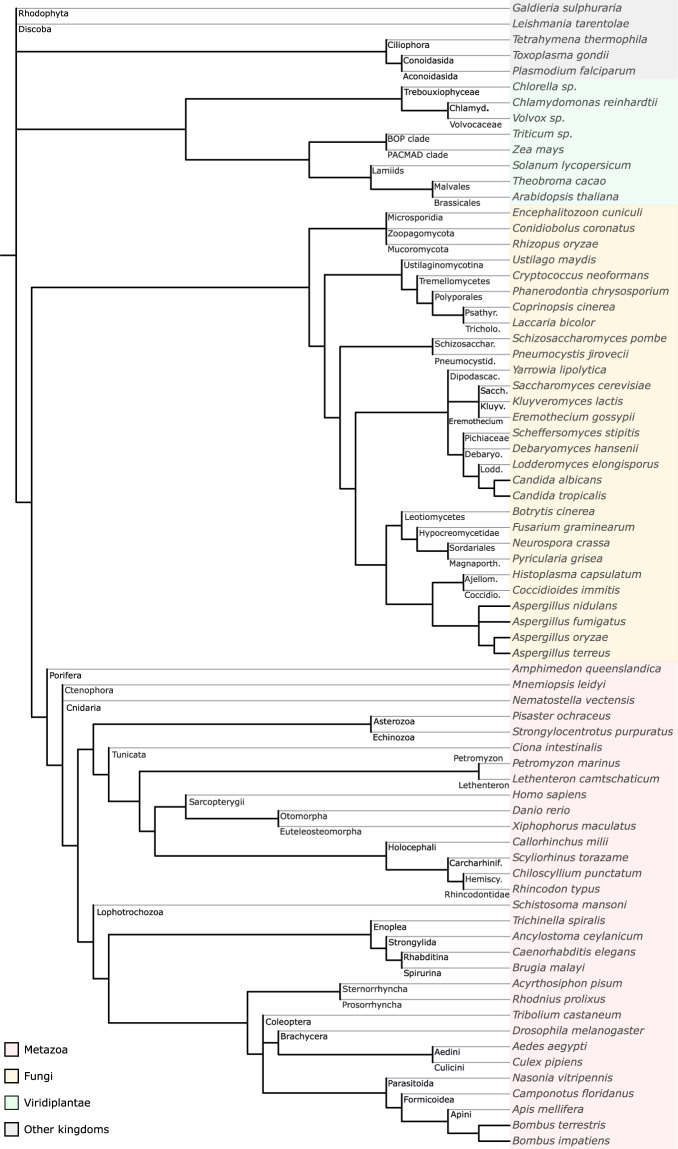
Phylogenetic tree of Augustus *ab initio* models showing the deeper taxonomic nodes used in the first step of the contig model selection.

For all contigs identified as eukaryotic by Kraken2, we used Augustus (v3.3), a software dedicated to *de novo* eukaryotic gene prediction^30^. The gene structure is complex in eukaryotes and changes across species^27^. Thus, Augustus provides *ab initio* models for 73 different species (Fig. 2) and one must be selected to perform gene prediction. Due to the conservation of genomic features across closely related organisms, we assigned, to each eukaryotic contig, a model based on its Kraken2 taxonomic annotation. Note that this model selection step does not aim at a definitive taxonomic annotation; here we used a sensitive approach to predict as accurately as possible putative eukaryotic genes that will then be filtered by a more selective homology-based taxonomic annotation approach at the protein level. Selection of the phylogenetically closest model for gene prediction on each contig was done using a custom python script^31^ which functions as follows:

- First, we browsed the 73 model species tree from the leaves to the root assigning a non-ambiguous parental taxonomic term to each model species as long as no bifurcation with a branch containing another model species was found (Fig. 2). For example, in plants, *Arabidopsis thaliana* is the sole representative of the Brassicales; so the Brassicales parental term was associated with the *A. thaliana* model. Consequently, we used the *A. thaliana* Augustus model for all eukaryotic contigs assigned with a taxonomic ID belonging to the Brassicales branch. Similarly, *Homo sapiens* is the only representative of mammals, so any contig identified by Kraken2 as a mammalian organism will be assigned the *H. sapiens* model.

At this point, an Augustus gene prediction model could be assigned to 7.1% (ca. 8 million) of contigs. The rest of the contigs (ca. 105 million) could not be assigned an unambiguous closest model species because they belonged to a bifurcating branch in the tree leading to several equally close model species.

**Fig. 3:**
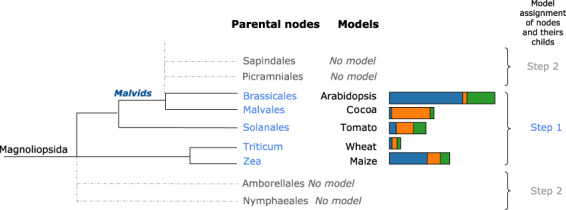
Phylogenetic tree focused on Magnoliopsida clades displaying the Augustus model distribution supporting the assignment of *ab initio* gene model by dataset (blue = Plant-associated, orange = Terrestrial 1, green = Terrestrial 2).

- Therefore, in a second step, for all these remaining eukaryotic contigs, we selected among the children branches the most frequently assigned model in the whole dataset the contig belongs to (i.e. Plant-associated, Terrestrial 1 or Terrestrial 2) at the previous step (first pass). To continue the previous example, the next more ancestral branch in the phylogeny of Brassicales is the clade ‘Malvids’ that displays a polytomy of eight children branches of which only two contain an Augustus model species (Malvales and Brassicales). Hence, no model could be unambiguously assigned to contigs with a Malvids taxonomic ID other than Malvales or Brassicales. Therefore, all contigs from other Malvids orders are processed with the most frequently assigned species model for each of the three datasets (Fig. 3). For example, they are processed with the cocoa gene model (Malvales, *Theobroma cacao*) in the dataset Terrestrial 1, or the *Arabidopsis thaliana* gene model in the Plant-associated and Terrestrial 2 datasets (Fig. 3). The distribution of contigs across the models is available in Supplementary Data, Fig. 1^32^.

Overall, our pipeline allowed assigning an Augustus model to ca. 101 million possibly eukaryotic contigs (Fig. 1). The most assigned ones were Metazoa and Viridiplantae models, with respectively 49% and 44% of contigs in plant-associated metagenomes and 76% and 16% in terrestrial data. In both datasets, we assigned fungal models to 6.5% of contigs (Supplementary Data, Table 1^33^); and the majority of other contigs were assigned to SAR, Discoba or Rhodophyta models. Although these last taxonomic groups were assigned at a relatively low proportion, this still corresponds to tens or hundreds of thousands of contigs. Unsurprisingly, the less assigned are gene models of aquatic animals such as some benthic animals, sharks, or also lamprey models. At this point, we could not assess whether the numerous assignments to metazoan and plant models came from mis-annotated contigs or contamination, therefore further analyses were performed after gene and protein prediction.

**Fig. 4:**
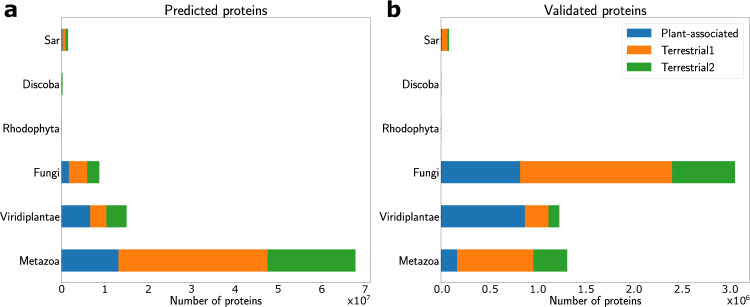
Number of Augustus-predicted proteins and their taxonomic distribution per Augustus model kingdom by dataset (**a**) on all contigs (**b**) on eukaryotic contigs validated by Diamond (blue = Plant-associated, orange = Terrestrial 1, green = Terrestrial 2).

Then, once a model species has been assigned to contigs we ran the eukaryotic gene predictor Augustus^30^, with default parameters, which allowed predicting 93 million protein-coding genes (Fig. 1). The number of proteins predicted per contig ranges from 1 to 410 with 2 protein predicted per contig on average for all datasets together. Consistent with the model assignment across kingdoms, the highest numbers of proteins were predicted for contigs assigned to Metazoan and Viridiplantae Augustus models. Moreover, we predicted 8.7 million proteins with Augustus fungal models and 1.8 million with different protist models (Fig. 4a).

### Confirmation of eukaryotic origins and improvement of the taxonomic information

To filter-out false-positive eukaryotic classification and assign a more reliable taxonomic annotation to the proteins predicted by our pipeline than simple inheritance from the Kraken2-based contig annotation, we used the last common ancestor algorithm of Diamond^34^. The homology search was run at the protein level with an E-value threshold of 10^−6^ and using the January 2020 release of the NCBI nr database^29^ as protein reference. In the LCA mode, Diamond will assign an NCBI taxonomic identifier (i.e. TaxID) based on the last common ancestor of all the hits with a score not diverging by more than 10% from the best hit score. Using an LCA approach constitutes a substantial gain in taxonomic annotation reliability compared to approaches based on the best BLAST hit alone, this single best hit being potentially mis-annotated itself, or sharing only low identity with the query sequence. This LCA approach is usually employed for taxonomic assignment of sequences distantly related to those of known organisms present in public sequence libraries, such as ancient or actual metagenomic data^28,35–38^. Consequently, the improved quality and completeness of protein sequences combined with a more accurate taxonomic assignment method is expected to yield a more reliable taxonomic annotation. From the 93 million proteins predicted by Augustus, 8,001,326 (present on 5,724,823 contigs from 4,083 metagenomes, Fig. 1) were assigned a eukaryotic taxonomic annotation by the Diamond LCA approach (Table 2) and are made available as a curated dataset of eukaryotic soil proteins^39^ with taxonomic informations^40^.

**Table 2:** Taxonomic classification of Augustus predicted proteins in superkingdoms by the Last Common Ancestor algorithm of DIAMOND among each dataset.

Clade	Plant-ass.	Terrestrial1	Terrestrial2	Total	%
Prokaryote	12,271,986	11,564,201	20,560,428	44,396,615	47.6
Eukaryote	4,986,024	1,951,235	1,064,070	8,001,326	8.6
Viruses	23,743	25,409	70,942	120,094	0.1
Undetermined	4,511,252	29,664,147	6,655,739	40,831,138	43.7
Total	21,793,005	43,204,992	28,351,179	93,349,176	100

**Fig. 5:**
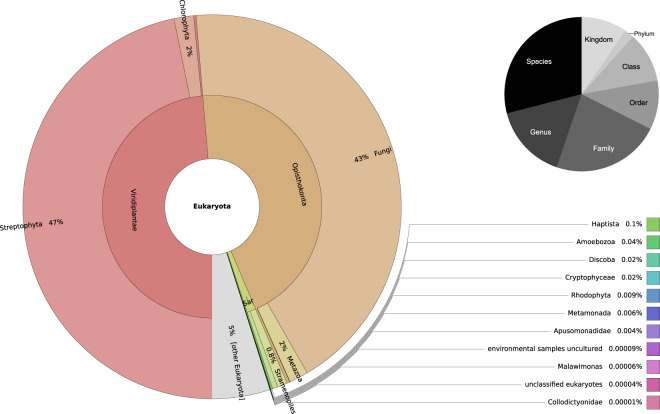
Krona representation of taxonomic assignment provided by the last common ancestor algorithm of DIAMOND for the 8 million eukaryotic proteins predicted by our homemade pipeline using Augustus (HTML file: available on Supplementary Data^42^), and the pie chart of taxonomic ranks of retrieved lineages.

Of these 8 million proteins, 45% were assigned a Opisthokonta taxonomy (Fungi + Metazoa), of which 96% were fungal and only 4% Metazoa (Fig. 5). These proportions are consistent with eukaryotic taxonomic distribution previously described in the literature, reporting fungi as the most abundant eukaryotic microorganisms in studied soil^2,41^. Actually, in soil metagenomes, fungal organisms are often second to bacteria in number and account for a comparable proportion of the biomass. Here, we retrieved 1,657 different fungal TaxIDs covering granularity levels ranging from species to the whole kingdom. Taxonomic annotations at deeper taxonomic nodes indicate the protein is equally related to proteins from multiple different and phylogenetically distinct fungal species. Among Metazoa, the dominant categories were Arthropoda, then Nematoda and Rotifera, respectively representing 48%, 9% and 8% of Metazoa (Supplementary Data^42^), again consistent with these species being the most abundant animals in soil environment. Besides Opisthokonta, Viridiplantae was actually the most represented kingdom, with 49% of all eukaryotic taxonomic assignment (Fig. 5). This suggests plant material is frequently present in soil samples and this is particularly expected for the plant-associated samples. Besides Opisthokonta and plants, other eukaryotes mainly belonged to the category SAR (1% of all) and most of the rest (5%) were unclassified eukaryotes (category other eukaryota, Fig. 5). These last taxa show small percentage of the whole dataset of soil eukaryotic proteins but still represent several thousand of proteins due to the size of the dataset.

The rest of the 93 million soil proteins were either assigned a non-eukaryotic TaxID with 47.6% and 0.1% being assigned a prokaryotic and viral taxonomy, respectively (Table 2), or had undetermined taxonomic annotation (43.7%).

### Identification of potential orphan eukaryotic proteins

More than 40 million proteins, representing 43.7% of the total Augustus predictions, could not be assigned a prokaryotic, eukaryotic or viral TaxID. Among them 27,269,572 (67%) were assigned untraceable taxonomic identifiers such as ‘unclassified’ (e.g. ‘12908’ TaxID) or ‘other’ (e.g. ‘32644’ TaxID), and the rest of the proteins (13,561,566 or 33%) simply returned no hit at all against the NCBI’s nr library in our Diamond homology search. Because these proteins might represent false positives from Augustus, they were not blindly added to the dataset of 8 million eukaryotic soil proteins. However, these proteins might as well represent orphan eukaryotic proteins lacking homology in public databases, constituting an important resource for new discoveries. To discriminate potential eukaryotic from non-eukaryotic orphan proteins, we assessed whether they were distributed on otherwise mostly-eukaryotic contigs. Thus, from the initial dataset of 113 million Kraken2-assessed eukaryotic contigs, we only retained orphan proteins present on contigs that contained at least 50% of Diamond-confirmed eukaryotic protein-coding genes. This yielded a total of 3,657,380 contigs distibuted on 4,059 metagenomes (Supplementary Data, Table 3^33^). A total of 354,243 orphan proteins were distributed on these contigs and represent potential novel eukaryotic proteins. We made this additional dataset of potential novel orphan eukaryotic proteins also available^43^.

**Table 3:** Data record, information about files available on public repository DATA INRAE^49^.

File name	Type	Size	Path	Description
eukaryotic_proteins.aa^39^	fasta	3GB	.	8 M of validated eukaryotic proteins predicted with Augustus in contigs from Terrestrial and Plant-associated metagenomic data from JGI
eukaryotic_proteins_taxonomy.txt^40^	text file	1,9GB	.	Taxonomic information for 8 M of validated eukaryotic proteins from the last common ancestor algorithm of Diamond
orphan_Euka.aa^43^	fasta	79MB	.	Orphan proteins from contigs with over half of eukaryotic proteins
eukaryotic_proteins_clustered.aa^45^	fasta	1.8GB	.	4,6 M representative clusters of 8 M of eukaryotic proteins
eukaryotic_proteins_clustered.tsv^47^	TSV	614MB	.	Composition of eukaryotic protein clusters
orphan_proteins_clustered.aa^46^	fasta	66MB	.	288,612 representative clusters of orphan proteins
orphan_proteins_clustered.tsv^48^	TSV	27MB	.	Composition of orphan protein clusters
eukaryotic_proteins_taxonomy_krona.html^42^	html	1,7MB	./Supplementary Data	Krona representation of 8 M of validated eukaryotic protein taxonomy from last common ancestor algorithm of Diamond
Supplementary_data_1.txt^24^	text file	158KB	./Supplementary Data	List of metagenome identifier of processed data from JGI
Supplementary_data_Figures.pdf^32^	PDF	323KB	./Supplementary Data	Fig. 1: Informations on eukaryotic proteins prediction processing
				Fig. 2: BUSCO scores by dataset
Supplementary_data_tables.pdf^33^	PDF	51KB	./Supplementary Data	Table 1: Kraken2 lineage distribution in main eukaryotic Clade
				Table 2: Number of proteins predicted with Augustus
				Table 3: Information on gene prediction outputs
				Table 4: Statistics of BUSCO scores

### Reducing redundancy of soil eukaryotic proteins

Some redundancy was expected because we used metagenomic data from thousands of individual studies, and some sequencing data came from the same sampling location. Therefore, we clustered Fasta files using the Linclust software of the MMseq2 metagenomic toolkit^44^. For both eukaryotic and orphan datasets, we clustered proteins with at least 99% sequence identity and covering at least 90% of the target. With these parameters, the 8 million eukaryotic proteins were clustered in 4,624,994 representative sequences^45^, and the 354,243 orphan proteins were clustered in 288,612 proteins^46^. For both clusterings, we provide the correspondence files to link original protein predictions to their respective representative clusters^47,48^.

## Data Records

All processed and Supplementary Data are publicly available on Data INRAE portal^49^ containing files described in Table 3.

## Technical Validation

### Comparison of protein prediction and taxonomic annotation quality to original JGI annotation

To determine whether using Augustus in our pipeline allowed improving eukaryotic protein predictions, we compared them to the predicted proteins obtained by the JGI using Prodigal for the same set of contigs. For this comparison, we used the same 3,657,380 contigs (covering 4,059 different metagenomes) containing at least 50% of predicted proteins with a eukaryotic taxonomy assigned by Diamond-LCA (defined above). Our pipeline allowed predicting 5.6 million proteins in these contigs. In comparison, on the same dataset, Prodigal initially predicted a total of 16 million proteins, covering 3,294,764 of these contigs and 3,979 metagenomes (Supplementary Data, Table 3^33^). First, although the number of protein predicted is higher with Prodigal, this software was unable to predict proteins in more than 360,000 contigs (3,657,380-3,294,764). Moreover, the raw number of proteins can be misleading because while Prodigal predicted 1,9 billion amino acids, our methodology allowed predicting 2.5 billion amino acids in total, suggesting although more proteins were predicted by Prodigal, they were much shorter and probably fragmented. Augustus allowed predicting introns in 1,627,033 genes from 1,074,415 contigs; these intronic sequences span on average 17% of the gene length. In comparison, Prodigal is not able to predict introns and ends its prediction when the first stop codon is encountered. Therefore, at least 28% (1.6/5.6 millions) of the proteins predicted by Augustus were necessarily incorrectly predicted by Prodigal, initially. Moreover with the high frequency of stop codons in the intronic regions due to less selective pressure on these genomic regions, most intron-containing genes are expected to be truncated by Prodigal. Overall, we oberve that our strategy was able to predict longer proteins and on more contigs that the initial Prodigal annotation. Hence, to further compare predictions from both methods, we used two metrics: *(i)* protein length distribution, and *(ii)* the recovery of nearly universal single copy eukaryotic genes.

#### Length distribution of protein

**Fig. 6:**
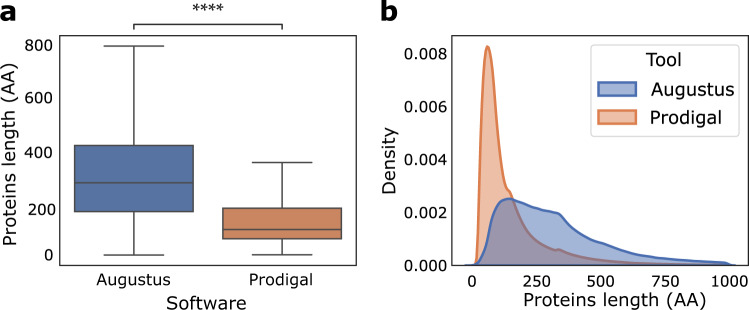
Distribution of protein lengths of Augustus prediction in blue versus Prodigal prediction in orange. Proteins from Augustus are significantly longer than those from Prodigal (see text).

First, we calculated and compared the distribution of protein lengths from Augustus vs. Prodigal predictions. Proteins predicted by Augustus were significantly longer than proteins predicted by Prodigal on the same contigs (Fig. 6; unpaired t-test, n = 5.3 10^6^/n = 9 10^6^ proteins, T = 1.994 10^3^, *p* ≤ 10^−4^). These observations coupled with the higher number of proteins predicted by Prodigal, confirm that Augustus was able to predict introns and join together multiple exons to form more complete genes where Prodigal predicted multiple truncated genes. Of note, the average size of genes (in ext. proteins) in eukaryotes is larger than in prokaryotes, due to the evolution of genome complexity^50^. Furthermore, the length distribution is closer to a normal one with Augustus predictions than with Prodigal ones (Fig. 6b), indicating a better quality of our new predictions. Indeed, Nevers *et al*.^51^ reports that a non-normal distribution of proteins length, as observed for these Prodigal predictions in eukaryotic contigs, is indicative of more truncated proteins caused by fragmented genomes and incorrect protein prediction. Overall, the authors showed that protein lengths distribution is remarkably well conserved across species and this feature could be used as quality metric in addition to other measures.

#### Recovery of nearly universal single-copy eukaryotic genes

To assess the improvement of our *de novo* eukaryotic protein predictions from soil microorganisms, we also compared the proportions of near-universal single-copy orthologs retrieved for each metagenome with those provided by Prodigal in the same contigs using BUSCO (v.4.0.2) in protein mode with ‘*eukaryota_odb10*’ lineage^52^. Starting from the 4,059 metagenomes containing contigs with at least 50% eukaryotic proteins, universally-conserved eukaryotic BUSCO proteins were identified in contigs coming from 1,093 metagenomes. This observation is not particularly surprising since *(i)* there are only 255 universally-conserved eukaryotic BUSCO genes, *(ii)* eukaryotes represent a minority of species in the soil^2^ and *(iii)* most eukaryotic genomes are only partially assembled from short-read based on shotgun metagenomic data.

**Fig. 7:**
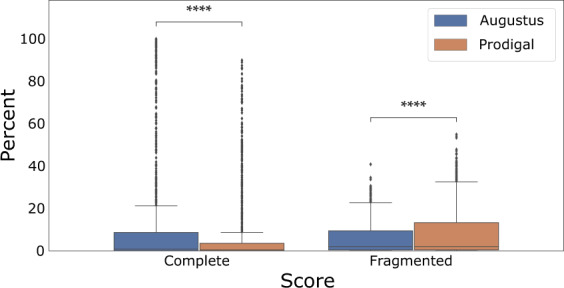
Complete and Fragmented BUSCO scores of the 1,093 metagenomes with single-copy universally conserved genes report a significantly better recovery of genes from eukaryotic microorganisms with Augustus than Prodigal (see text).

The proportion of BUSCO genes found in complete length in metagenomes was significantly higher for the Augustus predictions than for the initial Prodigal predictions (Fig. 7; paired Wilcoxon-test, n = 1,093 metagenomes, Complete T = 1.132 10^5^, p ≤ 10^−4^; Fragmented T = 2.039 10^4^, p ≤ 10^−4^). Similarly, the proportion of fragmented and missing BUSCO genes were significantly lower in Augustus predictions as compared to Prodigal predictions; this trend is identical for all datasets (Supplementary Data, Fig. 2^32^). BUSCO completeness scores from our Augustus gene predictions are as good or better than Prodigal for more than 98% of metagenomes. Furthermore, we have predicted more universal single-copy genes than Prodigal for 574 metagenomes, or more than half of the 1,093 metagenomes containing at least one BUSCO gene in one of both predictions. We observe an average improvement of 11.9% in the BUSCO completeness score, and genes are less fragmented in 510 metagenomes with an average of 8.5% lower proportion of fragments (Supplementary Data, Table 4^33^). The scores provided by BUSCO for these 1,093 metagenomes show a significant improvement of protein recovery and completeness for proteins from our Augustus-based strategy as compared to those from Prodigal, indicating our pipeline has improved the quality of eukaryotic gene models in soil metagenomes.

**Table 4:** BUSCO scores and FASTA files information for several gene prediction methods (1) Augustus with a mixture of model as in our paper, (2) Augustus with Fusarium model, (3) Augustus with Zebrafish model, (4) MetaEuk with NR database, (5) MetaEuk with Swissprot database and (6) Prodigal. All scores are computed on the same metagenome used as reference.

	Model	BUSCO scores					Fasta informations		
		Complete	Complete Single	Complete Duplicated	Fragmented	Missing	Nb. of Proteins	Total nb. of AA	Nb. of AA/protein
1	Mix	100	12.9	87.1	0	0	63,986	25,941,958	405
2	Fusarium	98.4	12.5	85.9	1.2	0.4	87,508	36,614,755	418
3	Zebrafish	96.1	23.9	72.2	3.1	0.8	152,796	43,294,314	283
4	Metaeuk nr	100	1.6	98.4	0	0	119,085	34,031,250	286
5	MetaEuk swp	97.6	8.2	89.4	0.8	1.6	34,906	12,112,481	347
6	Prodigal	77.3	36.9	40.4	20	2.7	271,456	37,520,032	138

#### Accuracy and diversity of taxonomic annotation

**Fig. 8:**
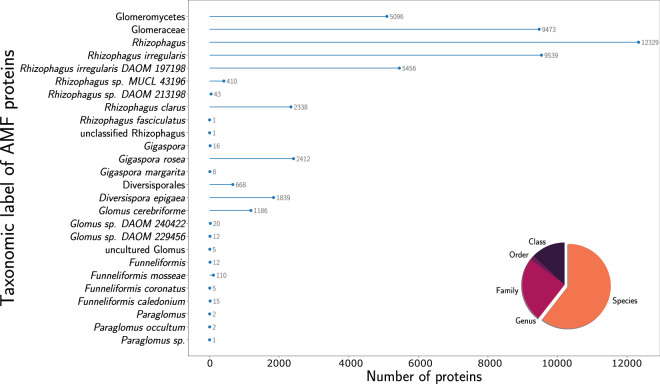
Annotated taxa of Arbuscular Mycorrhizal Fungal proteins with the last common ancestor algorithm of Diamond after protein prediction with Augustus. Number of proteins is shown for each taxa. The ratio of the taxonomic rank of annotations across AMF lineages is shown in a pie chart.

We assessed whether the Diamond-LCA taxonomic annotation strategy we employed allowed gaining information over the original JGI taxonomic annotation. To perform this evaluation, we compared the richness of taxonomic information proposed by our strategy to the original JGI annotation on a group of eukaryotic soil microorganisms known to play important ecological roles, Arbuscular Mycorrhizal Fungi (AMF). Indeed, AMF are ubiquitous members of soil microbiota, and more particularly of the (plant-associated) rhizosphere^15^. These eukaryotic microorganisms are plant symbionts with high impacts in several fields, mainly in agronomy due to a bio-stimulant and a bio-protective effect^16,53^, but they are also used to help in environmental issues such as cleaning-up polluted soils or facilitating reforestation. Among all the contigs containing at least 50% of eukaryotic proteins, according to Diamond-LCA, only 8,065 AMF proteins were predicted in the original JGI annotation, covering 6,048 contigs from 327 metagenomes. Moreover, all these proteins were assigned the same and sole AMF species/TaxID: *Rhizophagus irregularis*. In contrast, using our eukaryotic-centred gene prediction and taxonomic annotation pipeline, we expand the identification of AMF to 50,999 proteins in 48,726 contigs from 1,102 metagenomes. Furthermore, this new annotation now covers 26 different taxa (from class to species) better representing the AMF diversity present in these soils (Fig. 8). The case of these pervasive eukaryotic microorganisms in the soil highlights the benefits of this work to improve the representation of eukaryotic organisms in public soil metagenomes^40^.

### Validation of taxonomic assignment and gene prediction strategy

#### Taxonomic assignment methods

Comparing the Kraken2-assigned Augustus models for gene prediction on contigs to the taxonomic assignment at the protein-level based on Diamond LCA, we observed substantial differences in the relative proportion of taxonomic groups (Figs. 4, 5). For instance, while metazoan Augustus models were assigned to 49 and 76% of contigs in plant-associated and terrestrial datasets, respectively, only 2% of the eukaryotic proteins were assigned a metazaon taxonomy via Diamond-LCA. Conversely, while fungal Augustus models were assigned to only 6.5% of contigs, fungi represented 43% of taxonomic assignments obtained by Diamond-LCA. These Diamond-LCA taxonomic assignments are more consistent with the expected dominant taxa in the soil and illustrate the interest of our two-steps strategy with the first sensitive step aiming at identifying as many putative eukaryotic contigs as possible and the second specific step aiming at assigning an as reliable as possible taxonomic annotation to the genes and proteins. Furthermore at a global level, of the 93 million proteins predicted on the contigs deemed eukaryotic according to Kraken2, only 8 millions could be confirmed as eukaryotic with Diamond-LCA. An explanation for this discrepancy between Kraken2 and Diamond-LCA taxonomic assignments may be the following. A substantial proportion of contigs were probably assigned a eukaryotic taxonomy by Kraken2 based on a low number of k-mer matching with the eukaryotic target. The proteins predicted on these contigs were not assigned a eukaryotic annotation by Diamond but either a prokaryotic or undetermined taxonomy. Applying a confidence score threshold to Kraken2 taxonomic predictions might have resolved part of these false positives but at the risk of augmenting the rate of false negatives, and thus missing many eukaryotic contigs. Because we wanted this first filtering step to be as sensitive as possible, we decided not to apply a stringent confidence test on Kraken2 and to rely on further Diamond-based LCA strategy for more accurate final taxonomic annotation at the protein level.

We also compared the original single best BLAST hit JGI strategy for taxonomic annotation to the Diamond-LCA taxonomic assignment we employed in this study. Using a single best BLAST hit strategy, all taxonomic annotations were necessarily at the species level, regardless of the other hits and regardless of the percent identity with the best hit. This strategy can be misleading, in particular if the taxonomic annotation of the best hit is erroneous or if the similarity is only distant and to a variety of different species with no jump in E-values. In contrast, using an LCA approach, we noticed that only less than 30% of the proteins are still annotated at the species level. This indicates the rest of the proteins have been assigned a deeper taxonomic rank (Fig. 5) because they matched multiple hits with similarly good scores. This re-assignment of taxonomic annotation to deeper, more ancestral level decreases the risk of making errors by assigning a very shallow and precise taxonomic annotation based on spurious or distantly related best BLAST hits. This situation is expected to be particularly frequent when annotating proteins from environmental samples returning only distant similarity to proteins present in reference protein libraries from cultured organisms^35^.

#### Assessment of gene prediction strategy

As mentioned in previous sections, taxonomic annotations at nucleic (contig) and protein scales are not necessarily consistent. This fact may cast doubt on Augustus model selection procedure. To evaluate our soil eukaryotic gene prediction strategy, we compared the quality of proteins obtained by a mixture of Augustus models selected by our pipeline with prediction using either only one Augustus model, chosen as (i) Fusarium (a fungal model retrospectively corresponding to the most represented taxon in the final Diamond-based taxonomic assignment), or (ii) Zebrafish (a metazoan model corresponding to a taxon with low chance to be actually present in soil contigs), or (iii) another gene prediction software, MetaEuk^54^ with NR database as a reference and finally (iv) MetaEuk with SwissProt database as a reference. These four strategies were used to predict proteins on Kraken2-assigned eukaryotic contigs within the same dataset: metagenome ‘3300031471’ from the terrestrial dataset. This metagenome was randomly chosen among those containing complete eukaryotic BUSCO genes and thus representing an easy reference to check whether our mutli-model Augustus approach was relevant compared to single-model or reference database approaches. We compared eukaryotic BUSCO scores as well as the number of predicted proteins and the number of amino-acids per protein. Concerning Augustus, we observed the best recovery of universally conserved genes using our procedure (mixture of phylogenetically assigned models) (Table 4; line 1,2,3). Hence, although fungi and in particular *Fusarium* were the most numerous taxa in soil metagenomes, a mixture of models chosen by our procedure allowed a better recovery of BUSCO proteins. Thus, despite a necessarily substantial portion of imperfect model assignments, due to discrepancy between *a priori* Kraken2 taxonomic assignment and *a posteriori* Diamond taxonomic confirmation, a mixture of models seem to yield better results than a single phylogenetically close model. This is probably due to complex nature of soil communities. In contrast, and as expected, assigning a fish model for this soil sample returned the lowest BUSCO completeness and the highest proportion of fragmented and missing proteins. Concerning MetaEuk, BUSCO results were as good as our mixture of models procedure, when using the NCBI’s nr library as a reference (Table 4; line 1,4,5). However, a comparison of protein lengths distribution suggested that, besides BUSCO proteins, MetaEuk protein predictions were globally shorter with more proteins, a lower average number of amino acids per protein and a lower median length (Table 4). We tried whether changing the reference library in Metaeuk would improve protein lengths distribution by using Swissprot instead of nr. Using Swissprot indeed improved protein length metrics although these metrics were not as good as for our procedure, and came at the cost of decreased BUSCO completeness (Table 4, line 5). Overall, it seems that Metaeuk is more sensitive than the multi Augustus model we selected as more proteins were predicted. However, these proteins are shorter and might either represent short actual proteins or fragments. Our strategy was to be permissive at the contig level but stringent at the protein level (e.g. not to search for proteic ‘dark matter’). Although erroneous annotations inherent to massive high-throughput *de novo* gene prediction approaches can remain on some eukaryotic contigs, using Augustus with a mixture of gene models seems to represent the optimal balance between recovery of complete BUSCO genes and prediction of the longest and less fragmented proteins besides BUSCO ones.

## Usage Notes

Current microbiology investigations are focused on addressing the factors shaping the structure of microbial communities. To drive the development of tomorrow’s biotechnology it is essential to understand biological pathways both at the organism level and at the inter-microbial relationships scale, for prokaryotic and eukaryotic organisms together. This dataset provides a more complete and comprehensive view of the pool of genes and proteins, genetic diversity and distribution of eukaryotic microbes in soil and plant-associated microbiomes. At the molecular level, the use of this data is relevant to address biological questions in both fundamental research on plant-microbe interactions and applied, agronomical research, such as the study of potential metabolic functions of telluric eukaryotes, or of the interaction pathways between microbial members of the community. At a broader scale, the more accurate taxonomic annotation provides an unparalleled opportunity to assess how microbial eukaryotes are distributed across the soil and plant-associated microbial-environments. As illustrated in the data validation section, this improvement of microbial eukaryote representation has allowed us to increase by a factor of six times the detection of the ubiquitous AMF species, which are of high agronomic and economic interest.

Any research involving study of soil eukaryotes from evolutionary research on gene flow and transfers within the biome to more translational research aiming at deciphering important soil functions and biochemical pathways will benefit from this improved dataset of soil proteins with more accurate taxonomic annotation. In addition, our data can be cross-referenced with the metadata provided by the JGI (downloadable from the IMG/M portal) which includes geo-tracking and a wealth of environmental, sampling and processing information on each metagenome. They can be linked to proteins and annotations by searching for the ‘metagenomeID’, as each protein name in our dataset has a nomenclature based on the following pattern: ‘contigName_metagenomeID.geneID’, to offer this possibility. On one hand, the ecological metadata provides an unprecedented potential to study the effect of the environment on community structures and to have a better, more comprehensive view on how external factors influence the eukaryotic soil microbial communities. On the other hand, metadata on sampling and processing could be useful to assess which parameters affect the diversity and sequencing of eukaryotes in metagenomes and help to shape future protocols. Moreover, in this study, we provided a fully documented pipeline and protocol available as python scripts from the detection of putative eukaryotic contigs to the *ab initio* model selection for Augustus gene prediction and further Diamond-based taxonomic annotation, that can be re-used to improve the annotation of eukaryotes on any microbiome data, including in other biomes than the soil.

## Data Availability

• Project name: EukaProt_in_PublicSoilMetag^31^ • Project home page: https://github.com/CaroleBelliardo/EukaProt_in_PublicSoilMetag.git • Operating system(s): Platform independent • Programming language: Python3 • Other requirements: Python3.8 or higher • License: License: GNU General Public License v3.0
